# Editorial: Methods and applications in exercise physiology

**DOI:** 10.3389/fphys.2022.970615

**Published:** 2022-07-22

**Authors:** Andrew P. Hunt, Gary W. Mack

**Affiliations:** ^1^ School of Biomedical Sciences, Faculty of Health, Queensland University of Technology (QUT), Brisbane, QLD, Australia; ^2^ Department of Exercise Sciences, Brigham Young University, Provo, UT, United States

**Keywords:** exercise testing, glucose monitoring, transcranial direct current stimulation (tDCS), change of direction, electromyography - EMG, body composition, abdominal circumference

## Introduction

Exercise physiology is pivotal in optimizing health and performance as it influences various aspects of life, from sports performance to clinical rehabilitation and occupational health. *Methods and Applications in Exercise Physiology* aimed to highlight the latest experimental techniques and methods relating to research and practice. In this series, we capture recent advances in measurement techniques for 1) cardiorespiratory exercise testing, 2) athletic performance, and 3) health monitoring.

## Cardiorespiratory exercise testing

Valid assessment of maximum oxygen uptake and ventilatory threshold have been hallmarks of cardiorespiratory exercise testing for assessing athletic performance. Investigating whether VO_2peak_ measured by a single graded exercise test (GXT) is representative of an individual’s true maximum capacity, Hebisz et al. found no appreciable differences with verification tests performed 15 min after the GXT (cycling) or on a separate day. The ventilatory threshold and respiratory compensation point are other common markers of cardiorespiratory fitness measured during GXTs. Kim et al. developed and validated an automated method to identify these inflection points objectively. Together, these studies help to improve the way we measure key indicators of cardiorespiratory fitness.

## Athletic performance

Improvements in athletic performance are synonymous with enhanced muscular strength and endurance. Recent research has sought to elucidate if non-invasive brain stimulation technology can promote these outcomes. Transcranial direct current stimulation (tDCS) induces focal and transient changes in cortical excitability by applying a low-intensity direct current to the scalp. In a systematic review, Hu et al. analyzed the results of twelve studies, concluding that although tDCS does not significantly improve upper limb muscle strength, significant improvements in upper limb endurance performance (especially on the non-dominant side) can be achieved. Alternatively, the study by Lu et al. shows that tDCS can significantly improve muscle strength and explosive force of the non-dominant knee. Taken together, tDCS shows promise in offering an avenue to improve indicators of muscular performance.

Simpler and more accessible approaches have also been proposed for measuring muscle activity and change of direction (COD) performance. While monitoring muscle activity has conventionally involved intramuscular electromyography (EMG), which is invasive and requires specialized expertise, Date et al. have shown that surface EMG of Brachialis muscle activity accurately reflects intramuscular EMG. As such, surface EMG offers a non-invasive, easy to perform, less uncomfortable technique for research and clinical practice. On the sports field, COD performance plays a pivotal role in match-winning situations. Among youth basketball players, Chen et al. demonstrated that 505 COD performance measured with the COD timer app could be used to accurately capture timing data, compared to more expensive timing gate methods. Confirmation that these less expensive methods are valid and reliable will enable a broader range of applications for these performance assessments.

## Health monitoring

To help individuals with diabetes or poor glucose control improve glycemic control, continuous glucose monitoring has increasingly been adopted as a novel and feasible tool. Zhang et al. examined the accuracy of a flash glucose monitoring device among overweight/obese persons during sitting and walking before, during, and after an individual’s postprandial peak. Although absolute glucose readings from the flash glucose monitoring device underestimated plasma glucose, its overall accuracy was clinically acceptable during postprandial sitting and walking conditions in overweight or obese young adults, highlighting the potential for this technique to have widespread health-monitoring implications.

Body composition assessment has been an important aspect of research in exercise physiology for decades. Assessment techniques range widely in expense, required expertise, and validity. In favour of developing low-cost and readily applicable measurement techniques, Potter et al. investigated whether an abdominal circumference-focused method of percent body fat estimation accurately represented dual-energy x-ray absorptiometry. Their large-scale study of participants from the US Marine Corps concluded that the abdominal circumference estimate of percent body fat provides a field expedient method to classify individuals for obesity prevention but was not suitable for research-grade assessments.

Overall, this special issue on the methods and applications used in exercise physiology showcases the many diverse advances being made to the techniques used in research and practice. Less expensive methods that require minimal expertise and can be easily applied in field settings will continue to enhance our ability to measure, understand, and enhance human health and performance.

